# Waste Products

**Published:** 1893-02-25

**Authors:** 


### Waste Products.

"Many a little makes a muckle" is a true old Scotch saying. This is perhaps better understood in almost any country than England. In Germany, the idea of collecting waste products for charitable purposes seems to have arisen, but though various hospitals (small institutions started by private benevolence) have at different times tried collecting waste articles as a means of raising funds, they have all, as far as is known, given it up, the gains not being commensurate with the expense and trouble involved. But on the other hand, there are, however, several institutions which collect such articles chiefly for the support of orphanages, even though they make very small sums in this way, with the sole exception of those which collect "cigar-ends," (to be ground into snuff). The few institutions which do this on a payable scale seem to have been started for the special purpose, and though the number of cigars smoked in Germany is enormous (owing to their cheapness, and to the fact that soldiers are not allowed to smoke pipes in the streets, pipe-smoking has almost entirely died out), they do not make much by the cigar-ends themselves. Boxes to collect the ends are placed in private houses, restaurants, public-houses, &c., and these all serve to advertise their respective institutions; odd coins, also, are often put into the boxes. Schoolmasters usually attend to the collecting and forwarding of the contents of the boxes. There is one institution, which collects all waste articles on an extensive scale; this is the well-known Colony for Epileptics ("Bethel") at Bielefeld. It is to this institution that the things collected by children are usually sent. They collect everything they can get; for instance, cigar-ends and boxes, corks, the metal round the tops of wine-bottles, &c., old papers, books, &c., rags, old clothes of every sort, boots, hats, &c., broken china, glass, scraps of metal, nibs, stamps (new or old) collections of metals, coins, birds, &c., old woollen things being particularly welcome. Old nibs are sold as old iron, and obviously the returns are not worth mentioning. The Director of the Colony states that up to August, 1892, two years from the date he began collecting, he received over six thousand parcels, some of them of a very large size, of waste things. It is especially requested that parcels under 10 lbs. weight should not be sent. Rags, clothing, and bedding are thoroughly disinfected immediately they are received. Such articles as can be used by the institution itself, such as books, are handed over to it, the others are sold; the total amount realized in the two years, including the value of the things used by the institution (12,000 marks, or about £1,200), amounted to between 24,000 and 25,000 marks. Although this sum is a large one, it is hardly from a financial point of view, a repayment for the very large amount expended; and such a collection can only be made to pay at all when, as in this case, the labour is unpaid. The epileptics sorting, repacking, &c., and the large space and sheds needed are also supplied free. It is an advantage, however, from the point of view that these waste products supply the epileptics with light and easy work, and those who are trying to start an Epileptic Colony in England might especially note the fact. The Director of Bielefeld somewhat pathetically mentions that he is obliged to draw his friends' especial attention to the fact that unless the parcels are sent "carriage paid," the "gifts" are decidedly illusory, the contents of the parcels being, as a rule, worth less than the cost of the carriage.

